# Dominantly inherited micro-satellite instable cancer – the four Lynch syndromes - an EHTG, PLSD position statement

**DOI:** 10.1186/s13053-023-00263-3

**Published:** 2023-10-11

**Authors:** Pal Møller, Toni T. Seppälä, Aysel Ahadova, Emma J. Crosbie, Elke Holinski-Feder, Rodney Scott, Saskia Haupt, Gabriela Möslein, Ingrid Winship, Sanne W. Bajwa-ten Broeke, Kelly E. Kohut, Neil Ryan, Peter Bauerfeind, Laura E. Thomas, D. Gareth Evans, Stefan Aretz, Rolf H. Sijmons, Elizabeth Half, Karl Heinimann, Karoline Horisberger, Kevin Monahan, Christoph Engel, Giulia Martina Cavestro, Robert Fruscio, Naim Abu-Freha, Levi Zohar, Luigi Laghi, Lucio Bertario, Bernardo Bonanni, Maria Grazia Tibiletti, Leonardo S. Lino-Silva, Carlos Vaccaro, Adriana Della Valle, Benedito Mauro Rossi, Leandro Apolinário da Silva, Ivana Lucia de Oliveira Nascimento, Norma Teresa Rossi, Tadeusz Dębniak, Jukka-Pekka Mecklin, Inge Bernstein, Annika Lindblom, Lone Sunde, Sigve Nakken, Vincent Heuveline, John Burn, Eivind Hovig, Matthias Kloor, Julian R. Sampson, Mev Dominguez-Valentin, On behalf of the Prospective Lynch Syndrome Database (www.plsd.eu) and The European Hereditary Tumour Group (www.ehtg.org)

**Affiliations:** 1 Department of Tumor Biology, Institute of Cancer Research, Oslo University Hospital, PO Box 4950, 0424 NydalenOslo, Norway; 2 Faculty of Medicine and Health Technology, Cancer Centre, Tampere University and Tays, Tampere University Hospital, Tampere, Finland; 3 Department of Gastrointestinal Surgery, Helsinki University Central Hospital, University of Helsinki, Helsinki, Finland; 4 Applied Tumor Genomics, Research Program Unit, University of Helsinki, Helsinki, Finland; 5 Department of Applied Tumour Biology, Institute of Pathology, Heidelberg University Hospital, Heidelberg, Germany; 6 Clinical Operation Unit Applied Tumour Biology, German Cancer Research Centre (DKFZ), Heidelberg, Germany; 7 Molecular Medicine Partnership Unit (MMPU), European Molecular Biology Laboratory (EMBL), Heidelberg, Germany; 8 Gynaecological Oncology Research Group, Manchester University NHS Foundation Trust, Manchester, UK; 9 Division of Cancer Sciences, University of Manchester, Manchester, UK; 10 Medizinische Klinik Und Poliklinik IV, Klinikum Der Universität München, Campus Innenstadt, 80336 Munich, Germany; 11 Center of Medical Genetics, 80335 Munich, Germany; 12 Hunter Medical Research Institute, University of Newcastle, New Lambton, NSW 2305 Australia; 13 Engineering Mathematics and Computing Lab (EMCL), Interdisciplinary Center for Scientific Computing (IWR), Heidelberg University, Heidelberg, Germany; 14 Data Mining and Uncertainty Quantification (DMQ), Heidelberg Institute for Theoretical Studies (HITS), Heidelberg, Germany; 15 Surgical Center for Hereditary Tumors, Academic Hospital University, Ev. Bethesda Khs Duisburg, Düsseldorf, Germany; 16 Genomic Medicine, The Royal Melbourne Hospital, Melbourne, Australia; 17 Department of Medicine, University of Melbourne, Melbourne, Australia; 18 Department of Genetics, University of Groningen, University Medical Center Groningen, Groningen, The Netherlands; 19 Centre for Psychosocial Research in Cancer, Health Sciences, University of Southampton, Southampton, UK; 20 Medical School, University of Edinburgh, Edinburgh, UK; 21 Department of Gynaecology Oncology, Royal Infirmary of Edinburgh, Edinburgh, UK; 22 Hirslanden clinic, St.Anna Str. 36, 6006 Luzern, Switzerland; 23 Institute of Life Science, Swansea University, Swansea, SA28PP UK; 24 Manchester Centre for Genomic Medicine, Division of Evolution Infection and Genomic Sciences, University of Manchester, Manchester, M13 9WL UK; 25 Institute of Human Genetics, Medical Faculty, University of Bonn, Bonn, Germany; 26 National Center for Hereditary Tumor Syndromes, University Hospital Bonn, 53127 Bonn, Germany; 27 Department of Genetics, University of Groningen, University Medical Center Groningen, Groningen, The Netherlands; 28 Gastrointestinal Cancer Prevention Unit, Gastroenterology Department, Rambam Health Care Campus, Haifa, Israel; 29 Medical Genetics, Institute for Medical Genetics and Pathology, University Hospital Basel, Basel, Switzerland; 30 Department of General, Visceral and Transplatation Surgery, University Hospital of Mainz, Mainz, Germany; 31 Lynch Syndrome & Family Cancer Clinic, Centre for Familial Intestinal Cancer, St Mark’s Hospital, London, HA1 3UJ Harrow UK; 32 Institute for Medical Informatics, Statistics and Epidemiology, University of Leipzig, 04107 Leipzig, Germany; 33 Gastroenterology and Gastrointestinal Endoscopy Unit, Division of Experimental Oncology, IRCCS San Raffaele Scientific Institute, Vita-Salute San Raffaele University, 20132 Milan, Italy; 34 Clinic of Obstetrics and Gynecology, Department of Medicine and Surgery, University of Milan-Bicocca, Fondazione IRCCS San Gerardo, Monza, Italy; 35 Soroka University Medical Center, Ben-Gurion University of the Negev, Beer Sheva, Israel; 36 Service High Risk GI Cancer Gastroenterology, Department Rabin Medical Center, Rabin, Israel; 37 Laboratory of Molecular Gastroenterology, IRCCS Humanitas Research Hospital, Parma, Italy; 38 Department of Medicine and Surgery, University of Parma, Parma, Italy; 39 Division of Cancer Prevention and Genetics, IEO, European Institute of Oncology, Fondazione IRCCS Instituto Nazionale dei Tumori, IRCCS, 20141 Milan, Italy; 40 Division of Cancer Prevention and Genetics, IEO, European Institute of Oncology IRCCS, 20141 Milan, Italy; 41 Ospedale di Circolo ASST Settelaghi, Università dell’Insubria, Centro di Ricerca tumori eredo-familiari, Varese, Italy; 42 Surgical Pathology, Instituto Nacional de Cancerologia, Mexico City, Mexico; 43 Instituo Medicina Translacional e Ingenieria Biomedica - Hospital Italiano Bs As. - CONICET, Buenos Aires, Argentina; 44 Hospital Central de las Fuerzas Armadas, Grupo Colaborativo Uruguayo, Investigación de Afecciones Oncológicas Hereditarias (GCU), Montevideo, Uruguay; 45 Hospital Sírio-Libanês, São Paulo, Brasil; 46 Hospital Universitário Oswaldo Cruz/Universidade de Pernambuco, Recife, Brazil; 47 Instituto de Ciência da Saúde da UFBA/NOB-Grupo Oncoclinicas, Salvador, Brazil; 48 Fundación para el Progreso de la Medicina y Sanatorio Allende, Córdoba, Argentina; 49 Department of Genetics and Pathology, Pomeranian Medical University, ul. Unii Lubelskiej 1, 71-252 Szczecin, Poland; 50 Faculty of Sport and Health Sciences, University of Jyväskylä, Jyväskylä, Finland; 51 Department of Surgery, Central Finland Health Care District, Jyväskylä, Finland; 52 Department of Surgical Gastroenterology, Aalborg University Hospital, Aalborg University, 9000 Aalborg, Denmark; 53 Department of Clinical Medicine, Aalborg University Hospital, Aalborg University, 9000 Aalborg, Denmark; 54 The Danish HNPCC-register, Hvidovre Hospital, Hvidovre, Denmark; 55 Department of Molecular Medicine and Surgery, Karolinska Institutet, 171 76 Stockholm, Sweden; 56 Clinical Genetics, Karolinska University Hospital, Solna, Sweden; 57 Department of Clinical Genetics, Aalborg University Hospital, 9000 Aalborg, Denmark; 58 Department of Biomedicine, Aarhus University, DK-8000 Aarhus, Denmark; 59 Centre for bioinformatics, University of Oslo, Postbox 1080 Blindern, 0316 Oslo, Norway; 60 Centre for Cancer Cell Reprogramming (CanCell), Institute of Clinical Medicine, Faculty of Medicine, University of Oslo, Oslo, Norway; 61 Faculty of Medical Sciences, Newcastle University, Newcastle upon Tyne, NE1 7RU UK; 62 Institute of Medical Genetics, Division of Cancer and Genetics, Cardiff University School of Medicine, Heath Park, Cardiff, CF14 4XN UK

## Abstract

The recognition of dominantly inherited micro-satellite instable (MSI) cancers caused by pathogenic variants in one of the four mismatch repair (*MMR*) genes *MSH2, MLH1, MSH6* and *PMS2* has modified our understanding of carcinogenesis. Inherited loss of function variants in each of these *MMR* genes cause four dominantly inherited cancer syndromes with different penetrance and expressivities: the four Lynch syndromes. No person has an “average sex “or a pathogenic variant in an “average Lynch syndrome gene” and results that are not stratified by gene and sex will be valid for no one. Carcinogenesis may be a linear process from increased cellular division to localized cancer to metastasis. In addition, in the Lynch syndromes (LS) we now recognize a dynamic balance between two stochastic processes: MSI producing abnormal cells, and the host’s adaptive immune system’s ability to remove them. The latter may explain why colonoscopy surveillance does not reduce the incidence of colorectal cancer in LS, while it may improve the prognosis. Most early onset colon, endometrial and ovarian cancers in LS are now cured and most cancer related deaths are after subsequent cancers in other organs. Aspirin reduces the incidence of colorectal and other cancers in LS. Immunotherapy increases the host immune system’s capability to destroy MSI cancers. Colonoscopy surveillance, aspirin prevention and immunotherapy represent major steps forward in personalized precision medicine to prevent and cure inherited MSI cancer.

## Introduction

Ten years ago, revised guidelines were issued for the clinical management of a group of dominantly inherited cancer predisposition syndromes occurring in adults and caused by inherited pathogenic variants of the four mismatch-repair (*MMR*) genes *path*_*MLH1, path_MSH2, path_MSH6* and *path_PMS2* [1, 2]. They were referred to collectively as Lynch syndrome (LS). At the time, penetrance and expressivities of pathogenic variants of the four genes were not well established.

During organogenesis different genes are inactivated in different tissues [3] and, although the functions of the *MMR* genes are not restricted to tissues or organs derived from embryonic endoderm it appears that these tissues and organs do not have adequate alternative repair systems to compensate for faulty *MMR* genes.

Except for infrequent brain tumors and osteosarcomas, LS cancers occur in the endoderm-derived lining of the stomach, large and small intestine, the pancreas, bile duct, urinary tract, prostate and endometrium [4, 5]. Ovarian cancer in LS is often of an endometrioid subtype indicating that these cancers may be derived from the cells similar to endometrial cancers [6, 7]. While the *EPCAM* gene itself is not a cause of LS, the *MSH2* promoter is transcriptionally silenced by deletions involving its 3’ region which result in a distinct expression of LS cancers [8]. LS cancers are characterized by loss of the wild type allele following somatic (second hit) mutation, leading to micro-satellite instability (MSI) [9]. MSI cells produce abnormal peptides (neopeptides) which are recognized and targeted by the host immune system [10]. It was originally assumed that adenomas were precursors to all colorectal cancers (CRCs) and removal of adenomas by colonoscopy was advocated in carriers of pathogenic MMR variants (*path_MMR*) to reduce the incidence of CRC [1]. It soon became apparent, however, that CRC incidence was not reduced as much as expected by colonoscopy in LS. This paper discusses new knowledge reported the last decade.

## Epidemiology

### The Prospective Lynch Syndrome Database (PLSD)

In 2012 the European Hereditary Tumor Group (www.ehtg.org), at that time denoted the Mallorca Group, [1] decided to compile information on follow-up of *path_MMR* carriers across multiple specialist centres to answer three questions:

1. To what degree does colonoscopy surveillance reduce CRC incidence in *path_MMR* carriers?
2. What is the penetrance and expressivity of pathogenic variants in each of the four LS-associated genes?
3. What is the survival of carriers when followed-up as recommended, to facilitate early diagnosis and treatment?

The initial results were published by Møller et al. [11–13]. A more extensive and detailed report confirming the results in the three first reports was recently published [14]. Below we suggest an interpretation of the findings from these and further studies that were triggered by the initial results, and of concomitant tumor biological, prevention and treatment studies and mathematic modelling of the carcinogenetic paradigms that may help to explain what we observe.

#### The PLSD methods

The PLSD database, it’s structure and the methods for producing the results have been described in detail elsewhere [15–18]. A randomized controlled trial including a control group of *path_MMR* carriers who would be denied recommended medical interventions was considered impossible. Therefore, we performed an open, prospective observational study. Independently, a complementary retrospective segregation analysis in LS families was performed by the International Mismatch Repair Consortium (IMRC) which enabled estimation of the CRC incidence before surveillance colonoscopy had been widely implemented [19]. The results confirmed that cancer incidence in *path_MMR* carriers was not increased significantly before 25 years of age.

Our intentions were to examine the accepted paradigms of carcinogenesis and the effects of interventions. In all studies, the results obtained reflect the parameters used for ascertainment and/or the assumptions made when considering the results. To avoid these biases, when designing the PLSD neither the ascertainment model nor the methods used to compile the results included any assumption on carcinogenesis or the effects of interventions. Instead, the methods involved an assumption-free description of the empirical information observed. Compliant with the reporting methods of cancer registries, cancers were scored as discrete events by organ and age, allowing events to be considered as the result of stochastic probabilities in a time dimension.

The results obtained from the PLSD data were therefore empirically observed cancer incidences and subsequent overall survival in *path_MMR* carriers who were subjected to follow-up including colonoscopy surveillance in expert hereditary cancer centres world-wide. There is no indication that methodological problems could have substantially confounded the main results. Here we provide a brief summary and an interpretation of the results published to date by the PLSD.

#### The PLSD results

##### Colonoscopy and incidence of CRC

Compared with published estimates of CRC incidence in former generations before colonoscopy was widely instituted, CRC incidence in LS patients subjected to regular colonoscopy surveillance was increased for *path_MLH1* and *path_MSH2* carriers, not reduced for *path_MSH6* carriers and possibly (but not significantly) reduced in *path_PMS2* carriers < 50 years of age [20].

##### Penetrance and expressivity

**Fig. 1:**
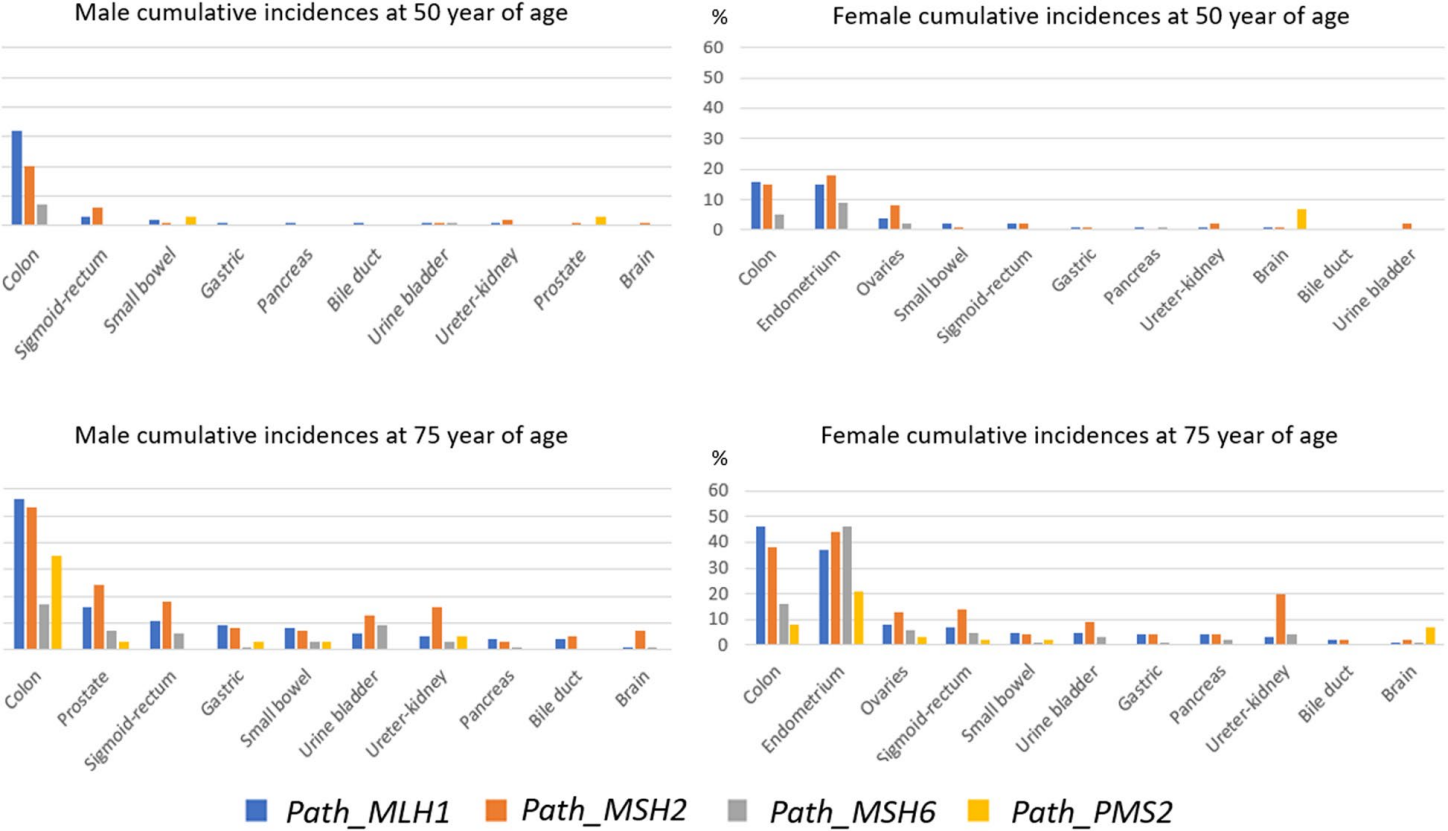
Cumulative incidences of cancers in male and female carriers subjected to colonoscopy stratified by gene, and sex and age (50 and 75 y.o.a.), ordered by incidence in path_*MLH1* carriers. The graphs are based on figures given in [14]

**Fig. 2:**
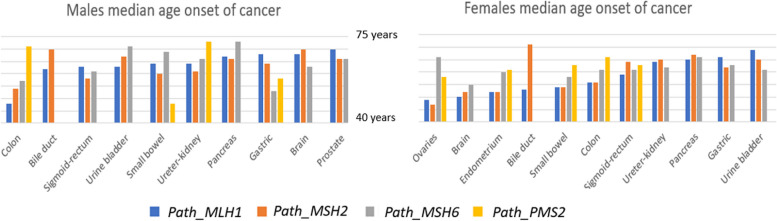
Median ages of onset of cancers in male and female carriers subjected to colonoscopy, by gene and sex, ordered by median ages in *path*_*MMR* carriers. The graphs are based on figures given in [14]

Cumulative cancer incidences stratified by MMR gene, sex, and carriers' age (50 and 75 y.o.a.) when subjected to follow-up, including colonoscopy to achieve early cancer diagnosis, are reported [14] and illustrated in Figs. 1 and 2. Cancers of the endometrium, colon and ovary started to appear in early adult life. Cancers of other organs were diagnosed later and mainly in survivors of earlier cancers [21]. Penetrance and expressivities were specific to each gene.

##### Survival after cancers detected during follow-up

**Fig. 3:**
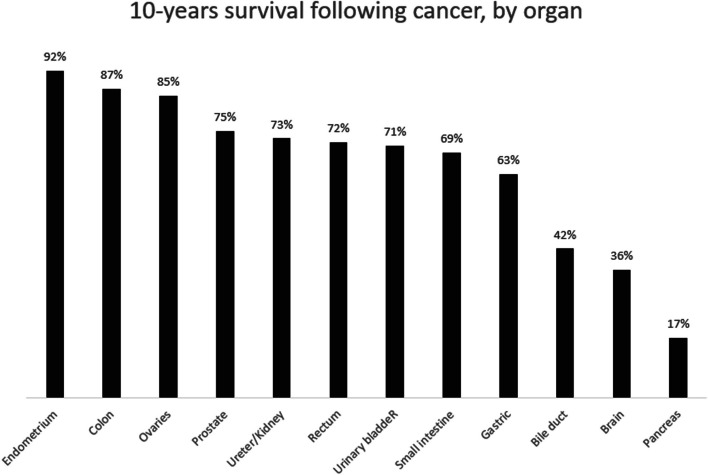
10-year survival following cancer in different organs in *path_MMR* carriers subjected to early diagnosis and treatment including colonoscopy. The graph is based on figures given in [14]. While there was no difference in survival between carriers of *path_MMR* variants by gene, cancer in *path_MSH6* and *path_PMS2* carriers were not frequent enough to measure survival apart from after endometrial cancer in *path_MSH6* carriers

**Fig. 4:**
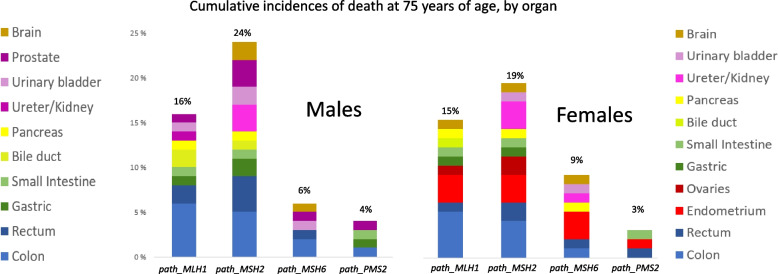
Cumulative incidence of death at 75 years of age following cancer in male and female carriers subjected to colonoscopy, by gene. The graphs are based on figures given in [14]

Most early onset cancers in the endometrium, colon and ovaries detected during follow up were cured [14] (Fig. 3). However, later in life, the survivors often developed cancers in other organs most of which were associated with lower overall survival [14] (Fig. 4).

These results indicated that both penetrance and expressivities of the genes in question have been affected by a substantial time-trend as most cases of CRC are now cured, which was not the case historically. In patients who received colonoscopy surveillance and relatively recent treatments (which in most individuals did not include immunotherapy), the results suggest that there are four different inherited MSI cancer syndromes:

#### The four Lynch syndromes

##### The *MSH2* Syndrome

The *MSH2* Syndrome is autosomal dominantly inherited. Penetrance is high.

*Path_MSH2* carriers are at high risk of cancers in all organs that are affected across the Lynch syndromes, with early onset of cancer in endometrium/ovaries and colon. Most carriers survive these first cancers following early detection and treatment. Cancers in other organs are most often diagnosed in survivors of the early onset cancers and include rectal, upper urinary tract, prostate and brain cancers.

Few founder variants – fitness is low

Most cancer deaths are associated with non-CRC cancers, particularly those of the endometrium, rectum, upper urinary tract, prostate and brain

Colonoscopy overdiagnoses colon cancer.

##### The* MLH1* syndrome

The *MLH1* Syndrome is autosomal dominantly inherited. Penetrance is high.

Early onset and high incidence of cancer in the colon, endometrium and ovaries. Most carriers survive their first cancer following early detection and treatment. Cancers in other organs are most often diagnosed in survivors of the early onset cancers, and most often include rectal, stomach, small intestine, bile duct and pancreatic cancers.

Few founder variants – fitness is low

Most cancer deaths are associated with non-CRC cancers, particularly those of the endometrium, bile duct and pancreas.

Colonoscopy overdiagnoses colon cancer.

##### The *MSH6* syndrome

The *MSH6* Syndrome is autosomal dominantly inherited with sex limitation. Penetrance is high in females but low in males.

There is a high incidence of endometrial and ovarian cancer that occurs at older ages than in *path_MSH2/MLH1* carriers. There is an increased incidence of CRC in both sexes that is nonetheless much lower than in *path_MSH2/MLH1* carriers. Incidences of cancers in other organs are low.

Few founder variants – fitness is low.

Detection rate by family history is low because of the sex-limited inheritance.

The inconclusive effect of colonoscopy is due to the low number of carriers reported to the PLSD.

##### The *PMS2* syndrome

The *PMS2* Syndrome is autosomal dominantly inherited.

*Path_PMS2* carriers have a slightly increased incidence of CRC and endometrial cancer in young adults, with higher incidence in older ages. Increased cancer incidence in other organs is not demonstrated.

Estimates of cancer incidences are difficult because of ascertainment biases.

Founder variants present, fitness seems good.

In contrast to the three other syndromes, the low incidence of CRC in young adults receiving colonoscopy surveillance may suggest that colonoscopy reduces CRC incidence.

Because of the very much lower cancer risks in the *PMS2* syndrome and potentially different carcinogenetic mechanisms associated with CRC development, the discussion below may not be pertinent to *path_PMS2* carriers.

### The PLSD and the InSiGHT variant database

When using the international InSIGHT database that indicates whether variants of the MMR genes are disease-associated or not [2], the PLSD database that indicates the penetrance and expressivity of pathogenic or likely pathogenic variants is displayed by selecting the MMR CANCER RISK tab. The PLSD is also available directly at www.plsd.eu. It interactively displays the remaining life-time risk for cancer in each organ when the user indicates the carrier’s age, sex and genetic variant. This feature of PLSD may be helpful to carriers and health care workers.

### Inherited congenital mismatch repair deficiency (CMMRD)

Inherited, biallelic *path_PMS2*, *path_MLH1, path_MSH2* and *path_MSH6* variants are recognized to cause the recessively inherited congenital mismatch repair deficiency (CMMRD) syndrome [22]. CMMRD is characterized by a high incidence of MSI non-endodermal malignancies in early life. *Path_PMS2* variants are the most frequent cause of CMMRD. The relative rarity of *path_MLH1* and *path_MSH2* causing CMMRD has caused speculation that most homozygotes or compound heterozygotes may not survive fetal development. A more detailed discussion of CMMRD is outside the scope of this paper.

### Relations in time between cancers and consequences of early diagnosis and treatment

Most of the frequent and early onset cancers in the four Lynch syndromes are cured following early diagnosis and treatment, which may be achieved in many cases through colonoscopy and gynaecologic examinations [13] and by promotion of cancer awareness with early consultation in relation to “red flag” symptoms. Most cancer-associated deaths in carriers who are subject to follow-up are now associated with non-CRC cancers [14].

Follow up studies suggest that the occurrence of a non-CRC first cancer was not associated with CRC incidence [12] and neither stage at CRC diagnosis, nor survival following CRC was associated with the interval between colonoscopies [23–26]**.** These findings were not as expected from the adenoma-carcinoma sequence paradigm of CRC. Instead, they were in keeping with the notion that the observed cancers are the result of stochastic probabilities in time, in conflict with the notion of linear progression from acquisition of an initial somatic pathogenic variant that causes increased mitotic activity, to a dysplastic adenoma and eventually an invasive and metastasizing cancer [27, 28]. If the stochastic model is correct for most LS cancers, the average incidences we have published are valid for groups but may have limited predictive value for an individual. No person has an “average sex “or a pathogenic variant in an “average Lynch syndrome gene” and results that are not stratified by gene and sex will be valid for no one. Modifiers of penetrance may be important in determining which cancers occur and when. More detailed analyses investigating whether there are associations between cancers, or whether they do indeed reflect stochastic chances, would be interesting.

Most cancers diagnosed before 50 years of age in male *path_MLH1* carriers are colon cancers, and cancers occurring in other organs reflect the time trend of increased colon cancer survival. Most cancers in young male p*ath_MSH2* carriers are also colon cancers, but rectal, urinary tract, prostate and brain cancers are more frequent later in life and with increased colon cancer survival they have become the major causes of death.

In females, however, endometrial and ovarian cancers together are the first and most frequent cancers in *path_MLH1* carriers and even more so in *path_MSH2* carriers. Colon cancer has a slightly lower incidence and older onset in *path_MSH2* than *path_MLH1* carriers. Considering LS as a syndrome of inherited CRC or gastro-intestinal cancers ignores gynaecological cancers as the main manifestation in women, and that gynaecological cancers together with urothelial, prostate and brain cancers are the leading causes of death in *path_MMR* carriers who receive follow-up with colonoscopy for early diagnosis and treatment.

### Ascertainment biases when estimating variant frequencies, penetrance and expressivities

Identification of LS families has been and still is biased. The first clinical criteria to identify affected families were constructed for research purposes to identify the causative gene(s) and reflected the misconception that we were looking for inherited CRC, not a syndrome of inherited cancer in many organs, and they primarily identified *path_MLH1* families [29]. The understanding that endometrial cancer was part of the syndrome was reflected in later criteria [30] and led to more *path_MSH2* and especially *path_MSH6* families being recognized. Because of sex-limited inheritance many *path_MSH6* families did not fulfil these clinical criteria which assumed high cancer incidences in both sexes [31]. *Path_PMS2* penetrance is so low that discrimination from normal variation by using family history is nearly impossible. A similar pattern of biases occurs when genetic testing is undertaken based on family history and when incident testing is focused on young onset CRC [32]. Only after all incidental cancers are tested for the four genes in question (in all ages and for all the genes in question) and the combined results are assembled, we will be able to fully determine the incidence and expressivities related to the genetic variants of the genes.

Contrary to the assumptions underlying the clinical Amsterdam 1 (AMS1) [29] and Amsterdam 2 (AMS2) [30] criteria, our studies in the PLSD have shown that ovarian cancer can be grouped together with endometrial and colon cancer as the early onset cancers in carriers who are subject to colonoscopy, while rectal cancer can be grouped with the other less frequent cancers that are seen mainly in survivors of the early onset cancers. Therefore, grouping colon and rectal cancer epidemiologically as one organ, as was done by the AMS1/2 criteria might now be considered a mistake. A possible interpretation of our findings is that colonoscopy may prevent rectal but not colon cancer in LS. The PLSD data also show that not including ovarian cancer in the AMS2 criteria was a mistake. And especially so when its treatment often includes hysterectomy which will prevent endometrial cancer. Including cancer in other organs will have little effect on the sensitivity of the criteria in the identification of LS families, because most of these cancers appear in survivors of the early onset cancers [21, 33].

Despite the consensus that colonoscopy reduces CRC incidence in the population as a whole, there is no recognized evidence that this statement is correct in *path_MMR* carriers, as there is only limited historical evidence based on three publications, all reporting observations made on a cohort of only 22 Finnish families [34–37].

The Finnish families were selected on the basis of one case of very early onset CRC with multiple CRC-affected relatives. Some of the additional methodological problems that were not discussed in those reports are as follows: the index clusters were not removed when calculating CRC incidences; it was incorrectly assumed that the family members had 50% carrier probability after the CRC-affected cases were excluded, and lead-time bias in the non-intervention group was not discussed. Therefore, the validity of the conclusions included in those reports is arguable. A later segregation analysis in 70 Finnish families of which 65 had a *path_MLH1* variant [38] reported higher CRC incidence than in French families [39] with *path_MLH1* variants, and much higher incidence than in a multi-national report on European families with *path_MLH1* variants [20]. Additionally, these Finnish papers described findings in carriers of the local Finnish *path_MLH1* founder variant which may not be representative of all *path_MLH1* variants and may not be representative for carriers of pathogenic variants of the other genes. Findings in a single series should be confirmed in another before they are used to support clinical decision-making. Despite the considerable time since the Finnish reports, there is no other reported evidence that colonoscopy reduces CRC incidence in LS [34].

### Ovarian MSI cancer

Ovarian cancer in LS carriers is dramatically different from ovarian cancer in *path_BRCA1/2* carriers [40]. The observed risk of dying from gynaecologic cancer diagnosed before 40 years of age for carriers of *path_MMR* variants was 0%, leading us to conclude that prophylactic hysterectomy and/or oophorectomy before 40 years of age solely for cancer prevention reasons is unwarranted and unethical. Similarly, the observed risk of dying from ovarian cancer in *path_MSH6* or *path_PMS2* carriers diagnosed before 50 years of age was 0%, and in these carriers, prophylactic oophorectomy before 50 years of age solely for cancer prevention reasons is considered unwarranted and unethical [41]. Etiologic diagnosis of ovarian cancer cases is crucial to select proper treatment for the affected individual, for planning their follow-up for subsequent cancers, and for cascade testing of their relatives when hereditary cancer is demonstrated.

## Combined results of epidemiological, biological, interventional and treatment studies

### Both the adenoma-carcinoma and stochastic non-linear paradigms are ‘true’

While the PLSD results indicate the assumption that removing colorectal adenomas would prevent most CRC in LS is wrong, they are not in conflict with the adenoma-carcinoma paradigm being true. Rather they indicate that the adenoma-carcinoma pathway is not the only pathway to CRC in LS. Based on currently available information, several possibly interacting carcinogenetic pathways may now be considered [10, 28]. Independent of the PLSD epidemiological studies, tumor biological studies and treatment studies in the last decade have provided new evidence for the mechanisms of colorectal carcinogenesis in *path_MMR* carriers. In addition to the linear adenoma-carcinoma paradigm, we now know that adult *path_MLH1* and *path_MSH2* carriers may at any time have a very large number of colonic crypts lacking normal *MMR* gene products (dMMR) which may develop directly into cancer without going through a macroscopically visible non-invasive tumor stage, such as an adenoma [42, 43]. These dMMR crypts may become MSI and immunogenic as they produce neo-peptides that are identified and attacked by the host immune system. The advances in MSI CRC treatment include immunotherapy which boosts the host immune system to eradicate MSI cells including invasive MSI cancers [44, 45], indicating that the host HLA system is a key barrier to cancer development [46]. Research on making cancer vaccines based on this biological understanding is ongoing [47, 48]. The information from concomitant biological and treatment studies is in keeping with the notion that invasive MSI cancers may be removed by the host immune system which may be in line with the epidemiological findings that frequent colonoscopy over-diagnoses CRC in *path_MMR* carriers.

### Consequences of the stochastic dynamic paradigm for carcinogenesis

The emerging combined picture of carcinogenesis in *path_MMR* carriers, is of a life-long dynamic situation in which carriers develop a very large number of MSI precursor lesions or tumors which are immunogenic and are controlled by the host immune system. A precursor lesion or tumor may escape by chance and become an invasive cancer. The observed frequency of this occurring may be used as a predictive probability for a group, but has low predictive value for a single individual. The epidemiological information indicates that such cancers might also be removed by the host immune system – a phenomenon which is demonstrated more dramatically when immunotherapy boosts the response and even advanced cancers may be destroyed. In summary, *path_MMR* carriers may manifest a life-long stochastic dynamic process of tumor initiation by the MSI pathway that is counteracted by the host immune system recognizing and destroying MSI tumors.

Overdiagnosis when screening for cancer as demonstrated by PLSD is not novel [49, 50]. While the mechanisms discussed here are specific for *path_MMR* carriers, there may be many and different mechanisms leading to over-diagnosis when screening is undertaken for cancers with different etiologies which all may involve a stochastic element, as discussed above.

### Modifiers of the stochastic probabilities

Aspirin reduces CRC cancer incidence in *path_MMR* carriers [51], probably by modifying carcinogenetic pathways [52, 53] or modulating the immune microenvironment. A recent paper reports resistant starch intake to be associated with an overall reduction in upper extracolonic cancers in *path_MMR* carriers unselected for gene or gender. The major effect was on upper GI cancers [54]. Immunotherapy which increases survival in cancer patients, may be considered an example of an environmental factor modifying the probability that the host immune system will identify and remove a cancer. Aspirin and immunotherapy may be considered environmental factors associated with preventing and curing cancers by modifying the probabilities for stochastic events occurring. Adenomas are not frequent in LS and apparently are not caused by pathogenic variants of the *MMR* genes. The concept that CRC starts with an adenoma that is later modified to become an MSI cancer, implies that adenomas modified by MSI cause CRC, not the other way around. This is illustrated in the very rare cases of sebaceous skin adenomas which may become cancers in *path_MMR* carriers (Muir-Torre syndrome) [55]. It is accepted that sporadic CRC is mostly caused by adenomas that by stochastic chance develop into CRC. The assumption that colonoscopy would decrease CRC incidence in LS was based on this concept [1]. In recent years, growing evidence from both epidemiological (the PLSD reports) and biological [10, 27, 28, 56] reports indicate that there is an additional, and perhaps more frequent carcinogenetic pathway to CRC in LS which starts with MSI caused by *path_MMR* variants and that may not include a non-invasive adenomatous stage before invasive cancer occurs. The existence of the adenoma-carcinoma pathway is no argument against the MSI pathway also being true, and *vice-versa*. Carcinogenesis is complex, there is no single model to explain it all.

Recognizing that colonoscopy as a modifier probably prevents most CRCs that arise via the adenoma-carcinoma pathway may indicate that in *path-MMR* carriers undergoing colonoscopy surveillance and polypectomy, the observed CRCs in carriers subjected to colonoscopy arise mostly via the MSI pathway without a macroscopically visible tumor. If invasive MSI cancers are destroyed immunologically by the host, the more frequently colonoscopies are carried out the more frequently CRC will be observed, because some CRCs observed would otherwise have been removed later by the host immune system. Theoretically, this may challenge the normal association between early detection/treatment and improved survival. This may, at least in part, be a consequence of colonoscopy blocking the adenoma-carcinoma pathway, while in LS the immune system might remove invasive cancers. This notion is supported by an early Finnish study which reported the survival in patients receiving three-yearly colonoscopies together with the treatment available at that time [38] and that found comparable survival to that observed by PLSD today [14]. This study, however, described carriers of the local Finnish *path_MLH1* founder variant which may not be representative of all *path_MLH1* carriers and almost certainly not representative for carriers of pathogenic variants of the other genes. The late progression to metastatic spread and the good prognosis when LS-associated CRCs are removed surgically [57], may reflect that MSI cancer cells are vulnerable to being identified and removed by the host immune system when they are located outside the primary MSI cancer’s local environment.

Although recent epidemiological studies indicate that surveillance colonoscopy in *path_MMR* carriers has not decreased CRC incidence as hoped for, prevention of CRC-associated death is its true objective and this is largely being achieved. The PLSD results indicate that colonoscopy surveillance should be continued, but question the benefit of colonoscopy being performed more frequently than every three years. Immunotherapy will hopefully increase survival following MSI cancers, including the later onset cancers with currently poor survival. Aspirin reduces MSI cancer incidence, and in future anti-cancer vaccination may so do as well.

In general, cancer incidences increase exponentially with increasing age. Why, in *path_MMR* carriers, there is close to zero increase in cancer incidence before 25 years of age and why cancer incidence does not increase substantially after 50 years is not understood. It may possibly be associated with maturation and ageing of the host immune system and its interactions with MSI cells [58].

## Are there any additional genes that cause inherited MSI cancers?

Variants of other genes may cause inherited MSI cancers through interactions with the four *MMR* genes described here. As mentioned in the introduction above, inherited deletion of the *EPCAM* tail causes silencing of the *MSH2* promoter [8]. The *POLE* genes variants may cause somatic mutations in the *MMR* genes which in effect may cause inherited MSI cancers [59].

## Different pathobiology of the two most common inherited cancer syndromes

The knowledge-based development of immunotherapy tailored to treat both inherited and sporadic MSI cancers, comes in addition to the knowledge-based development of PARP inhibitors to treat both inherited and somatic *BRCA1/2* deficient DNA double-stranded break-associated cancer [60].

Because about 15% of all CRCs are MSI and may benefit from immunotherapy, there is a growing consensus that all CRC cases should be tested for MSI [61]. Germline testing for LS may be restricted to patients with MSI cancers and time-consuming family history documentation may become less important for other cases, enabling genetic counsellor time to be focused on those with proven germline predisposition and their relatives.

We now have knowledge-based personalized precision treatment for the two most frequent inherited cancer groups, and for biologically similar but sporadic cancers. All individuals with these types of cancer will benefit from testing to determine which carcinogenetic mechanisms have caused their cancer, enabling the selection of the most appropriate treatment, follow-up for subsequent cancers when needed, and for cascade testing in their families. Ovarian cancer, which is common to both *path_MMR* and *path_BRCA1/2* carriers, and where both non-inherited MSI and *path_BRCA1/2* associated cancers occur, is an example.

## Conclusions

This paper identifies, delineates and denotes the group of dominantly inherited Lynch syndromes by their shared inherited trait which is MSI cancers (https://www.genome.gov/genetics-glossary/Mendelian-Inheritance). Corresponding with OMIM (https://www.ncbi.nlm.nih.gov/omim/?term=LYNCH+SYNDROME), we recognize four different Lynch syndromes caused by the four *MMR* genes, as described in this paper, and consider 3’ *EPCAM* deletions only as an alternative mechanism by which *MSH2* may be silenced. The numbering of the Lynch syndromes in OMIM is confusing and in conflict with how Henry Lynch grouped and numbered them. Instead, we name the distinct syndromes by the four genes that cause them which is precise and self-explanatory, and gives room for more groups to be named in future if more genes are found to cause MSI cancers.

No person has an “average sex “or a pathogenic variant in an “average Lynch syndrome gene” and results that are not stratified by gene and sex will be valid for no one. The penetrance and expressivities of the genetic variants causing the Lynch syndromes are age-dependent, and discussions of penetrance or expressivity without considering age gives limited information. Average ages at which cancers are diagnosed reflect the ages of cases ascertained, not necessarily the ages at which cancers usually occur. Reports that are not stratified on genetic variants, sex and age may have limited utility. Compiling series with enough carriers to consider age, gene and sex needs wide international collaboration, as has been achieved by the PLSD.

Improved survival following early diagnosis and treatment of MSI cancers of the colon, endometrium and ovary has led to carriers living on and contracting subsequent cancers in other organs. These have worse prognoses. Tests for MSI have generally been optimised to demonstrate MSI colon cancers, but the different Lynch syndrome genes have different organ specific penetrance and expressivities. The prevalence of MSI in cancers in these other organs is not well studied, with respect to either how to test for MSI cancers in these organs or to estimate the frequency of MSI cancers. Identifying such MSI cancers would be of interest to select cases for immunotherapy tailored against MSI cancers. The obvious next steps for clinical research on the *MSH2* and *MLH1* syndromes include determining the effects of immunotherapy for cancers with currently poor prognoses.

When the *MMR* genes were identified as the causes of the Lynch syndromes, it was assumed that colonoscopy and removal of pre-invasive adenomas would prevent colon cancer. However, despite wide implementation of these measures, reduction in colorectal cancer incidence by colonoscopy surveillance has not been documented. Colonoscopy should be advocated to improve the prognosis when colon cancer is diagnosed, not to reduce colon cancer incidence in the Lynch syndromes.

Colon cancer may be asymptomatic and MSI colon cancer typically spreads late. Reports on colon cancer incidence that have not controlled for lead time bias should be interpreted with caution.

The host immune system may remove invasive MSI cancers, explaining overdiagnosis of colon cancer when colonoscopy surveillance is undertaken. A randomized control trial of colonoscopy versus no colonoscopy would not be possible for ethical reasons. What we could and should do, is to conduct a trial of one year versus three years intervals between colonoscopies and measure colon cancer incidences and survival. The quality of colonoscopy is subjected to time-trends. Whether improved techniques will reduce MSI colon cancer incidence remains to be seen.

Tumours are mostly tested genetically for structural gene changes and less commonly for epigenetic silencing, despite knowledge that the somatic “second hit” needed to abrogate mismatch repair is often epigenetic. Most MSI colon cancers are sporadic and result from bi-allelic somatic epigenetic silencing of an MMR gene. It may be of interest to discover more about the mechanisms causing second hits that lead to cancer in *path_MMR* carriers, and MSI sporadic cancers in non-carriers. These factors may be stochastic events, but they may also have inherited genetic components.

## Data Availability

Not applicable.
